# PFTAIRE peptide degrading cyclin Y and ameliorating cognitive deficits in mouse models of Alzheimer’s Disease

**DOI:** 10.1002/alz.088711

**Published:** 2025-01-09

**Authors:** Illhwan Cho, Minhae Cha, Hye Yun Kim, YoungSoo Kim

**Affiliations:** ^1^ Yonsei Institute of Pharmaceutical Sciences, Incheon Korea, Republic of (South); ^2^ Yonsei University, Incheon Korea, Republic of (South); ^3^ Yonsei University, Incheon, Incheon Korea, Republic of (South); ^4^ Yonsei Institute of Pharmaceutical Sciences, Incheon, Incheon Korea, Republic of (South)

## Abstract

**Background:**

Cyclin Y (CCNY) is a member of cyclin protein family inhibiting long‐term synaptic plasticity, which is related to the learning and memory function in neuronal system. Recently, CCNY has been reported to associate with the cognitive deficits in Alzheimer’s disease (AD).

**Method:**

In this study, we discovered PFTAIRE peptide to diminish CCNY protein level and to ameliorate cognitive dysfunction in AD. This peptide is derived from the domain of cyclin‐dependent kinase 14, a kinase degrading CCNY protein, and this domain is essential for the binding to CCNY. Utilizing solid‐phase peptide synthesis, PFTAIRE peptide was produced.

**Result:**

We observed that PFTAIRE peptide binds to CCNY protein in *in vitro* studies. We confirmed the plasma and liver microsomal stability of the peptide and the ability to penetrate blood brain barrier. We intravenously injected PFTAIRE peptide to AD mouse models and performed behavior tests. PFTAIRE peptide prevents memory impairments in AD mouse models. In the proteomic analysis of the cortical brain section of mouse models, we observed the alteration of 93 proteins by the PFTAIRE peptide compared to non‐treated groups. Utilizing gene ontology, PFTAIRE peptide up‐regulated proteins related to release/transport neurotransmitter in brain synapse and down‐regulated proteins related to oxidative stress.

**Conclusion:**

Overall, the discovery of PFTAIRE peptide suggests new approach for the development of therapeutics targeting AD.

